# Field Report of the Singapore Emergency Medical Team Deployment Following the 2025 Myanmar Earthquake: Clinical and Operational Insights from a WHO Type-1 Fixed Facility

**DOI:** 10.1017/S1049023X25101593

**Published:** 2025-12

**Authors:** Mo Hom Nang, Guek Gwee Sim, Patricia Sueh Ying Lee, Shu Fang Ho, Evelyn Swee Kim Boon, Ahmad Khairil Bin Mohamed Jamil, Kee Chong Ng, Joy Quah

**Affiliations:** 1. Singapore General Hospital, Singapore; 2. Changi General Hospital, Singapore; 3. Sengkang General Hospital, Singapore

**Keywords:** disaster medicine, Emergency Medical Team, humanitarian aid

## Abstract

On March 28, 2025, a 7.7-magnitude earthquake struck the Sagaing region of Myanmar, resulting in 3,816 deaths and 5,104 injured, with Mandalay Region sustaining the most severe damage. Singapore Emergency Medical Team (SGEMT), verified by the World Health Organization (WHO) in 2024 as a Type-1 fixed Emergency Medical Team (EMT), was deployed in response. This mixed-methods study reports on the patient case mix and operational challenges encountered during the deployment. Data were derived from daily situation reports, clinical health records consistent with the WHO minimum data set (MDS), post-deployment review proceedings, and unstructured interviews with administrative, clinical, and logistics leads.

Deployment was delayed by diplomatic complexities and logistical challenges in freight transport. Clinical operations commenced on April 8, 2025 at Bahtoo Stadium, Mandalay, where SGEMT managed 1,803 patients over eight days. Quantitatively, 21.6% presented with direct earthquake-related injuries, 7.9% with conditions indirectly related to displacement, and 70.5% with chronic or unrelated conditions, reflecting patterns observed in other post-earthquake responses. Acute respiratory infections were the predominant infectious disease. Most patients were female, underscoring the importance of gender-sensitive approaches. The integration of a physiotherapist in a Type-1 facility, beyond WHO EMT minimum standards, enhanced clinical efficacy and rehabilitative capacity.

Qualitatively, thematic analysis guided by the 4Cs of disaster partnering –coordination, cooperation, communication, and collaboration – revealed critical enablers and constraints within the Association of Southeast Asian Nations (ASEAN) humanitarian framework. Findings highlight the need to reinforce regional coordination mechanisms to strengthen future disaster response in complex geopolitical situations.

## Introduction

On March 28, 2025 at 12:50h local time, an earthquake of magnitude 7.7 struck Sagaing region of Myanmar.^1^ The official reported death toll was 3,816 and 5,104 injured, with Mandalay Region being the hardest hit where one-half of all buildings were reported as either severely damaged or destroyed.^2,3^

That evening, the Myanmar State Administration Council (SAC; Naypyidaw, Myanmar) declared a state of emergency and appealed for international aid.^4^ The Singapore Emergency Medical Team (SGEMT), composed of 34 civilians, responded to the call for aid. Under the Emergency Medical Team (EMT) initiative, SGEMT was accredited by World Health Organization (WHO; Geneva, Switzerland) in September 2024 as a Type-1 fixed team. As follows, SGEMT has three branches: administrative, clinical, and logistics.^5^ The clinical branch consisted of six doctors, 11 nurses, one physiotherapist, and one psychologist. The administrative branch consisted of five members, and the remaining ten members were logistics personnel.

Emergency Medical Teams are categorized based on mobility and the level of care provided.^6^ Type-1 EMTs provide daylight hour care for stabilization of acute trauma and non-trauma cases with referral to in-patient and primary care network. Type-2 EMTs provide general and obstetric surgical care in addition to Type-1 services. Type-3 EMTs provide intensive care and complex referrals and interdisciplinary care.

This study seeks to examine the deployment of a Type-1 fixed EMT in response to the 2025 Myanmar earthquake, with a focus on clinical analysis and the operational challenges encountered.

## Sources and Methods

This mixed-methods field report is based on four data sources: (1) daily situational reports during deployment; (2) handwritten SGEMT patient health records collated into end-of-day tally sheets electronically, corresponding to the minimum data set (MDS) record defined by the WHO;^7,8^ (3) meeting minutes of a post-deployment after-action review meeting with all members; and (4) unstructured interviews with the administrative, clinical, and logistics leads of SGEMT.

Quantitatively, descriptive analysis was performed for patient demographics and clinical characteristics, and results were expressed as frequencies and percentages. Qualitatively, a deductive thematic analysis was performed, guided by the 4Cs of disaster partnering framework: coordination, cooperation, communication, and collaboration.^9^ Coders were given clear definition of each category. Coding was performed by JQ and NMH within the four pre-defined categories and AK resolved any disagreements. All four sources were used to triangulate information.

Ethical approval was obtained from Singapore Health Services Centralized Institutional Review Board (ref: 2025-0898).

## Observations

The Myanmar SAC appealed for international aid on March 28, 2025, the evening of the earthquake strike.^2–4^ The Association of Southeast Asian Nations (ASEAN) Coordinating Centre for Humanitarian Assistance on Disaster Management (AHA Centre; East Jakarta, Indonesia) was in-country the following day.^10^ On March 31, SGEMT registered with the AHA Centre for deployment and the formal request to deploy was issued on April 2. On the night of April 3, SGEMT departed Singapore for Yangon, Myanmar. The following day, the team undertook a one-hour domestic flight to Naypyidaw, followed by a 270-kilometre overland transfer through the towns of Yamethin and Meiktila, enroute to Mandalay City. These two towns were initially designated as deployment locations; however, these plans were twice altered upon arrival due to updated coordination instructions from the AHA Centre and the Department of Disaster Management, Myanmar (Naypyidaw, Myanmar). The 37-tonne freight was transported from Singapore to Yangon using four commercial cargo flights conducted between April 4 and April 5. They were loaded onto a series of trailer trucks and transported approximately 600 kilometers to Mandalay City by road. The changes in deployment location added an additional 100 kilometers of land transfer and contributed to freight delay.

Between April 5 and April 6, SGEMT built its clinical base of operations on a football field within Bahtoo Stadium. The stadium sits 900 meters away from Mandalay City’s largest hospital – Mandalay General Hospital (MGH). A splinter group visited MGH, a 1,500-bedded tertiary hospital, to develop a referral system for SGEMT under the direction of the local health authorities. At the time, MGH was functioning at markedly reduced capacity post-earthquake due to severely damaged infrastructure.^11^ Parking lots and service roads were transformed into temporary in-patient wards. In the 72-hour period after the earthquake, the emergency department of MGH experienced an attendance exceeding 500 patients per day. Patients requiring intensive care management were transferred to Kandawnadi Hospital, approximately six kilometers away. These two hospitals became the main referral centers for SGEMT.

**Figure 1.**
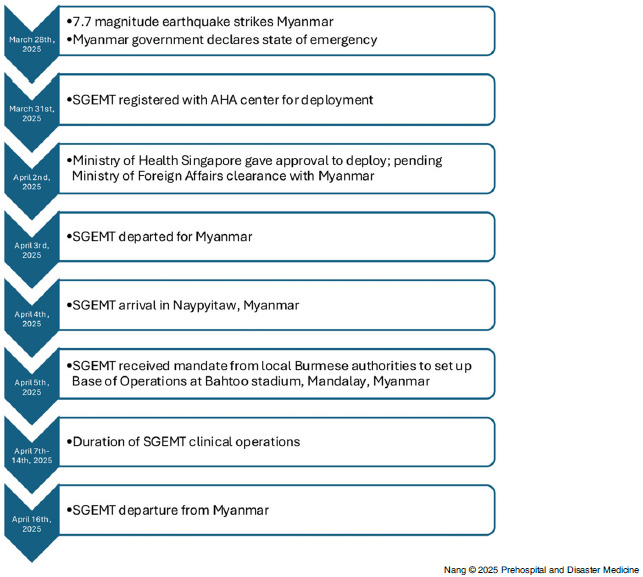
SGEMT Deployment Timeline. Abbreviations: SGEMT, Singapore Emergency Medical Team; AHA, ASEAN Coordinating Centre of Humanitarian Assistance on Disaster Management.

On April 7, approximately ten days after the earthquake and within 48 hours of site allocation, SGEMT began clinical operations. Figure 1 shows a detailed timeline. In total, SGEMT attended to 1,803 patients over a period of eight consecutive days. The descriptive data are presented in Table 1. In accordance with WHO MDS definitions, 21.6% were diagnosed with injuries as a direct consequence of the earthquake, such as infected wounds, fractures, and sprains; 7.9% of cases were indirectly related to the earthquake, including skin diseases and acute respiratory infections from having to stay in crowded, temporary makeshift tents due to displacement. The remaining 70.5% had conditions which were unrelated to the earthquake. The most common diagnosis on the WHO MDS tally was “other diagnosis” (67.9%) which is a diagnosis other than those related to acute trauma, infectious diseases, emergencies, skin diseases, acute mental health problems, obstetric complications, and severe acute malnutrition. Examples of such conditions include osteoarthritis, sciatica, peripheral neuropathies, poorly controlled hypertension, or diabetes. The second most common diagnosis was minor injury (17.9%), followed by acute respiratory infections (4.5%). The top three infectious conditions were acute respiratory infections, acute watery diarrheal diseases (0.9%), and acute bloody diarrheal diseases (0.2%). There were no recorded deaths or births. Majority of the patients (96.7%) were discharged. For patients requiring transfer, ambulances were provided by the Myanmar Red Cross Society (MRCS; Naypyidaw, Myanmar). Within the scope of allied health services, the clinical psychologist provided care to 46 patients, and the physiotherapist managed a caseload of 221 patients, prescribing a total of 72 sets of walking assistive devices. There were more female (68.6%) patients. Those with gender-specific health concerns reportedly preferred consultation with female health care providers.

**Table 1.** Demographic and Clinical Characteristics of Patients Presenting to SGEMT

Category	n (%)
**Gender**	
Female (Non-Pregnant)	1,155 (64.1)
Female (Pregnant)	81 (4.5)
Male	567 (31.4)
**Age**	
Pediatric	
<1-Year-Old	2 (0.1)
≥1 to < 5-Years-Old	45 (2.5)
≥ 5 to <18-Years-Old	94 (69.7)
Adult	
≥ 18 to <65-Years-Old	1,257 (69.7)
Geriatric	
≥ 65-Years-Old	405 (22.5)
**Triage Category**	
P1	34 (1.9)
P2	65 (3.6)
P3	1,704 (94.5)
**Health Conditions** ^a^	
Trauma	
Minor	323 (17.9)
Moderate	50 (2.8)
Major	8 (0.4)
Infectious Diseases	
Acute Respiratory Infections	82 (4.5)
Acute Watery Diarrhea	17 (0.9)
Acute Bloody Diarrhea	3 (0.2)
Emergencies (Medical/Surgical)	34 (1.9)
Skin Diseases	59 (3.3)
Acute Mental Health Problems	46 (2.6)
Severe Acute Malnutrition	7 (0.4)
Obstetric Complication	2 (0.1)
Others ^b^	1,225 (67.9)
**Etiology of Health Condition**	
Directly Related to Earthquake	389 (21.6)
Indirectly Related to Earthquake	143 (7.9)
Unrelated to Earthquake	1,271 (70.5)
**Disposition**	
Discharge without Medical Follow-Up	1,343 (74.5)
Discharge with Medical Follow-Up	405 (22.5)
Discharge Against Medical Advice	4 (0.2)
Referral with Transfer	51 (2.8)

Abbreviations: SGEMT, Singapore Emergency Medical Team; WHO, World Health Organization; MDS, minimum data set.

a Not all 22 health conditions listed in the WHO EMT-MDS daily reporting form are reflected in this table. Only selected conditions with relevance to the article have been displayed.

b Diagnosis which does not fall into any of the above categories or subcategories.

Local health authorities recommended medical students and displaced MGH staff as translators, while SGEMT had the support of an ethnic Burmese medical officer to facilitate discussions with local health authorities.

## Analysis

Quantitative clinical data showed that most patients, as seen in other post-earthquake EMT deployments, had chronic conditions unrelated to the acute event.^12,13^ This usually becomes more evident after day ten.^14,15^ For SGEMT, this pattern may also be attributed to the team’s delayed arrival, by which time the acute trauma burden had likely diminished substantially. This highlights the need for EMTs deploying past the initial phase to be able to manage large caseloads of chronic, non-trauma, and non-communicable conditions in disaster settings, where they also provide continuity of care due to disruption of local health services and infrastructure. This suggests a role for inclusion of primary care physicians and increased supply of chronic medications in future planning given the significant chronic disease burden. With regards to infectious diseases, the top diagnosis was acute respiratory infections, which differs from literature where gastrointestinal and skin diseases tend to be more common.^16^

There were disproportionally more female than male patients. Evidence consistently demonstrates that women are more likely to seek medical attention following disasters, often due to heightened vulnerability to injury, psychosocial distress, and disruptions in access to reproductive and maternal health services.^17–20^ Future EMTs should include female health professionals, psychosocial support, and reproductive health services, while ensuring privacy and culturally appropriate communication.

The WHO EMT minimum technical standards for rehabilitation for a Type-1 fixed facility does not currently include having a rehabilitation specialist.^21^ Early rehabilitative specialist access is linked to better functional outcomes and community reintegration, and SGEMT’s inclusion of a physiotherapist improved clinical efficacy by sharing the rehabilitative load.^22–24^

To date, no published studies describing EMT responses to the Myanmar earthquake are available for comparison. In a similar Type-1 fixed medical response to an earthquake in Türkiye 2023, Maddah, et al reported similar findings.^12^ Their deployment similarly began one week after the acute event, but it lasted longer, up to three months after. The top three diagnosis were identical and similar in percentages, with 72% presenting with “other diagnosis,” 12% diagnosed with minor injuries, and 9% with acute respiratory infections – the top infectious disease encountered. There were also more females than males seeking medical attention.

The four themes that directed the deductive thematic analysis of SGEMT operations originate from the 4Cs of disaster partnering, and refers to coordination, cooperation, communication, and collaboration.^9^ Coordination refers to alignment of actions of organizations to achieve a common objective, in this case, to deliver humanitarian aid to the affected population. The WHO EMT Coordination Cell (EMTCC) is recognized as the global leader in overseeing the entire life cycle of EMT deployment, from initial request and arrival to their departure and debriefing, together with the host country. This is described in detail by Takada, et al in response to the 2015 Nepal earthquake.^25^ However, the intricate diplomatic context in Myanmar precluded the involvement of WHO as a coordinating authority. The AHA Centre was allowed certain coordination rights, in conjunction with the Department of Disaster Management, Myanmar. Diplomatic constraints affecting central coordination, coupled with freight delays due to the closure of Mandalay International Airport, likely contributed to the delay in SGEMT’s site confirmation and operational setup. It is, however, recognized that a formal mandate from local authorities is essential for effective deployment, even if it results in some delays. Proceeding without authorization can diminish operational effectiveness, regardless of EMT preparedness.

Cooperation refers to short-term, informal, and voluntary relationships between organizations. To mitigate the absence of WHO EMTCC, the AHA Centre advised all EMTs to liaise directly with the local authorities and hospitals to build a referral system.^26^ Ultimately, SGEMT’s local referral network worked for three main reasons. Firstly, the endorsement of SGEMT referrals by the local health authorities. Secondly, early, open, face-to-face dialogue between SGEMT and the referral hospitals built a working relationship and enhanced familiarity with each other’s capabilities. Thirdly, SGEMT’s location in the city center was within a short distance of these tertiary centers. This model shows that locally sourced referral networks may suffice for optimal patient care, as an alternative. However, there may have been a possibility of further relieving the burden on the local health facilities by referring suitable patients to international Type-2 or Type-3 EMTs within the city instead, favoring a regional EMT coordinating body familiar with EMT typologies and capabilities. Transfer ambulances and crew were provided by MRCS at short notice, initiated via a three-way cooperation between SGEMT, Singapore Red Cross Society, and MRCS. Recently, ASEAN has signed a memorandum of understanding with International Federation of the Red Cross & Red Crescent Societies (Geneva, Switzerland) on the strengthening of community resilience in Southeast Asia, which includes disaster relief and emergency response.^27^ These existing close working relationships within the same humanitarian field allows for cooperation and interoperability. While SGEMT only requested for ambulances, more than 20 MRCS volunteers turned up to assist with queue management and area maintenance. This ties in with the observation by Grimm, et al post-Cyclone Nargis (2008) where strong social capital and volunteerism as a form of performing civic duty contributed strongly to health system resilience.^28^ These volunteers gave feedback to SGEMT staff that active volunteering gave them a sense of purpose and allowed them to process their grief and health together as a community.

Communication was vital at two distinct levels: with the local authorities and hospitals, in addition to between clinicians and patients. The key issue identified in meeting with local authorities and hospitals was the lack of familiarity with WHO EMT typology. Initially, there was resistance from the local authorities to issuing Bahtoo Stadium as there were reservations that a stadium could function as a field clinic and that SGEMT was largely self-sufficient enough to care for hundreds of patients daily. Having an ethnic Burmese speaker within SGEMT ensured that information was effectively conveyed, respecting cultural nuances. Deploying bilingual health care workers native to the affected country may be useful in deployments to overcome communication barriers and foster trust with the local community. Communication between clinicians and patients was bridged by medical students and health care staff from MGH.

Lastly, collaboration is characterized by a long-term relationship between organizations (or nations) with high levels of interdependency to solve joint problems with power symmetry. In contrast to the Cyclone Nargis in 2008, where the appeal for international aid only came three weeks post-event, Myanmar has demonstrated willingness for international collaboration by appealing within hours.^29,30^ Like Cyclone Nargis, EMTs from ASEAN nations were welcomed, but nations beyond were carefully considered and selected. To successfully mitigate global geopolitical tensions which may affect disaster relief work in Southeast Asia, the AHA Centre, as an ASEAN entity, has and must continue to shoulder the enormous task of being the lead collaborator and EMT coordinator in the region, by tapping on regional trust.^31^ This is in line with the AHA Centre’s mandate and ASEAN’s goal of being a global leader in disaster management.^32,33^

## Conclusion

The SGEMT earthquake relief deployment in Myanmar demonstrates that Type-1 EMTs must be prepared not only for acute trauma, but also for chronic conditions, gender-specific needs, and rehabilitation. Strengthening coordination, cooperation, communication, and collaboration within ASEAN, utilizing the WHO EMT framework, will be critical to ensuring resilient and timely humanitarian responses in future regional crises.
